# A Research Program on Implementing Integrated Care for Older Adults with Complex Health Needs (iCOACH): An International Collaboration

**DOI:** 10.5334/ijic.4160

**Published:** 2018-05-09

**Authors:** Walter P. Wodchis, Toni Ashton, G. Ross Baker, Nicolette Sheridan, Kerry Kuluski, Ann McKillop, Fiona A. Miller, John Parsons, Timothy Kenealy, On behalf of the iCOACH study team

**Affiliations:** 1 University of Toronto, CA; 2 University of Auckland, NZ

**Keywords:** protocol, implementation, integrated care, Community Based Primary Health Care, older adults, Innovative Models of Care, chronic conditions, complexity, case study

## Abstract

Health and social care systems across western developed nations are being challenged to meet the needs of an increasing number of people aging with multiple complex health and social needs. Community based primary health care (CBPHC) has been associated with more equitable access to services, better population level outcomes and lower system level costs. Itmay be well suited to the increasingly complex needs of populations; however the implementation of CBPHC models of care faces many challenges. This paper describes a program of research by an international, multi-university, multidisciplinary research team who are seeking to understand how to scale up and spread models of Integrated CBPHC (ICBPHC). The key question being addressed is “What are the steps to implementing innovative integrated community-based primary health care models that address the health and social needs of older adults with complex care needs?” and will be answered in three phases. In the first phase we identify and describe exemplar models of ICBPHC and their context in relation to relevant policies and performance across the three jurisdictions (New Zealand, Ontario and Québec, Canada). The second phase involves a series of theory-informed, mixed methods case studies from which we shall develop a conceptual framework that captures not only the attributes of successful innovative ICBPHC models, but also how these models are being implemented. In the third phase, we aim to translate our research into practice by identifying emerging models of ICBPHC in advance, and working alongside policymakers to inform the development and implementation of these models in each jurisdiction. The final output of the program will be a comprehensive guide to the design, implementation and scaling-up of innovative models of ICBPHC.

## Background

Health and social care systems across western developed nations are being challenged to meet the needs of increasing numbers of older adults with co-existing, multiple chronic conditions, the risks of which are exacerbated for socially and economically vulnerable populations [1]. International trends as well as health services and policy literature suggest that effective treatment that meets the clinical, social and cultural needs of older people with chronic and complex illness is best provided by integrating care across a multi-sectoral continuum including primary and specialized medical care and home and community care. Providers also need to be skilled and committed to providing seamless transitions between services for their clients with closer communication between services [2,3]. Within this context, community-based primary health care (CBPHC) providers are striving to offer innovative models of care that meet the complex needs of this diverse population group [4,5].

Promising approaches to chronic condition management in localised contexts are well represented in the literature. The Chronic Care Model (CCM) [6] in particular, is often used as a guide for system re-design for better primary health care for people with chronic disease [7,8,9]. The CCM is based on six essential elements that are fundamental to effective partnerships between informed, activated patients and prepared, proactive practice teams: 1) community resources and policies; 2) health care organization; 3) self-management support; 4) delivery system design; 5) decision support; and 6) clinical information systems. However, the focus of chronic care management is often centred on the management of single diseases for individuals rather than on the provision of population-based, comprehensive and integrated health and social care services for people with multiple conditions and their families [1,10,11,12,13,14]. In this study we use the CCM to understand and characterize the delivery of ICBPHC.

Ideal models of integrated CBPHC (ICBPHC) are comprehensive, person-oriented, inclusive of carers and family, health promoting, strengths-based, and without a singular disease focus. They also address problems of inequity in health and risk across population sub-groups. Specific cases of ICBPHC have been implemented internationally, including in New Zealand [15] and in some of Canada’s provinces [16,17,18] demonstrating better health outcomes, and reductions in costly and often inappropriate hospital and residential long-term care. However the spreading and scaling up of these models of care is generally weak and many initiatives have proved unsustainable. Internationally, no models appear to have reached scale that encompasses the population of a healthcare system [19]. As a result, there is considerable inequity in accessibility of ICBPHC for individuals with multiple complex health needs. It is therefore important to gain a better understanding of what makes ICBPHC possible and successful in different contexts, and to develop strategies for scaling them up to other populations or implementing them in other jurisdictions [20,21,22,23,24]. The implementation of ICBPHC requires policy, organizational, provider and patient-oriented approaches [25].

Implementation frameworks emphasise the multiple levels [21,22,26,27] and steps in the process [28,29] as well as the influence of the environmental context on how well new practices are adopted and sustained [30,31,32]. This paper describes a program of research by an international, multi-university, multidisciplinary research team that utilizes these frameworks to examine the implementation of ICBPHC in New Zealand, and in Ontario and Québec, Canada. The particular value of international and cross-jurisdictional research is in exploiting the diversity of approaches and outcomes achieved in the face of similar problems and issues. This approach also allows us to make comparisons both within and across jurisdictions.

## Research question

The key question being addressed is “What are the steps to implementing innovative integrated community-based primary health care (ICBPHC) models that address the health and social needs of older adults with complex care needs?”

The objectives of the programme are:

- To characterize and describe innovative models of ICBPHC and their contexts
- To develop a conceptual framework for the implementation of ICBPHC
- To understand the attributes of effective, innovative integrated models of ICBPHC
- To assess the degree to which such models address the needs of older adults with complex needs and their families/whānau (New Zealand Māori term for extended family)
- To assess how innovative ICBPHC delivery models address the needs of complex patients and their families/whānau
- To assess how organizational factors and inter-organizational relationships affect implementation in local contexts; and
- To assess how key dimensions of health policy affect the development, implementation and transferability of models from one context to another.

## Design and Methods

**Figure 1:**
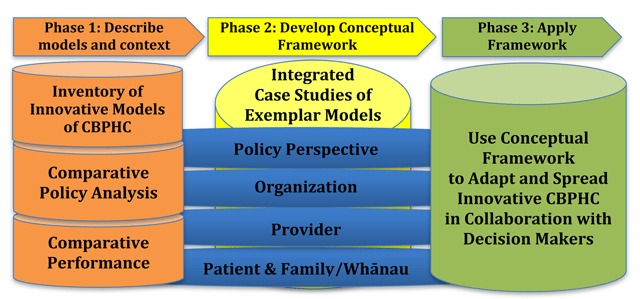
Phases of Research.

The program of research encompasses three phases (outlined in Figure 1). The research program began in 2014 and we are currently in the midst of the second phase. In the first phase we used one year to identify and described specific cases of ICBPHC and their context in relation to relevant policies and performance in each of the three jurisdictions of Ontario, Québec and New Zealand. The second phase is undertaken over two years and involves a series of theory-informed, mixed methods case studies from which we shall develop a conceptual framework that captures not only the attributes of successful innovative ICBPHC models, but also how these models are being implemented. In the third phase, we aim to translate our research into practice by identifying emerging models of ICBPHC in advance, and working alongside policymakers to inform the development and implementation of these models. It is anticipated that this phase will require two years for completion. The final output of the program will be a systematic guide to the design, implementation and scaling-up of innovative models of ICBPHC.

Research methods include reviews of literature, official documents and databases and mixed methods case studies (including surveys, interviews, and analysis of administrative data). Knowledge translation and capacity building is a foundation for the research program. We have developed this program from four perspectives: policy, organization, provider and patient and family/whānau.

## Phase One: Describe models and context

### Inventories of current cases

The research team developed inventories of specific cases of ICBPHC for older adults in New Zealand, Ontario and Québec by searching published peer reviewed and grey literature, the websites of government agencies and ICBPHC organizations, and by consulting with decision-makers and other health sector colleagues. A combination of literature searches (of empirical and gray sources) and stakeholder engagement guided the selection of cases to study, with the latter providing the most fruitful method. We conclude that it is possible to use personal networks and experts exclusively. It is not clear how much value formal searching adds over and above expert advice. However in a situation where there is no existing definitive list of potential cases, and no acknowledged “gold standard” way to create such a list, it seems appropriate to gather cases using multiple methods and to document those methods systematically (see Kuluski et al., 2017 in this collection) [33].

### Case Selection

From these inventories, three specific cases were selected in New Zealand, three in Ontario and one model implemented in three settings in Québec for the case study analyses of Phase Two. We refer to these cases as exemplars because they are representative of different successful approaches to implementing integrated care, not because they are necessarily perfect or ideal examples. Criteria for selection included the integration of primary care and home-and community based services, diverse populations and a patient-oriented approach to chronic care management (excluding cases with disease-based approaches). We also selected a range of models of care that also represented different phases of implementation, including early as well as more established initiatives and that were not time-limited pilot projects.

Details of the cases are provided by Breton et al. (2017) [34], a brief overview is provided here. In New Zealand, cases included 1) a Māori-led (New Zealand indigenous people) rural/regional service that includes medical and nursing care, home visits, shared care plans with a focus on individuals and whānau (extended family), self-management support, telehealth monitoring, and referrals to other social, cultural and community, and health services; 2) a rural/regional model with case-management, holistic care of individual and family, lifestyle coach for wider health and social/family needs, multi-disciplinary integrated care; 3) a regional multi-disciplinary service including hospital, primary health care and home-based services, rehabilitation, self-management, extended medical services in community, health promotion. In Ontario, cases included 4) an urban community health centre serving a Chinese immigrant population that focusses on a range of services: primary care, health promotion, housing services, legal services, coordination/referral/partnership with other providers/agencies including home care, education, immigrant settlement, social services; 5) an urban centre that provides for community support (home care, transportation, meals), access to primary care (medical and nursing), and coordination/referral to other community support agencies (rehabilitation); 6) an urban partnership with an interdisciplinary team inclusive of home care care-coordinators and members of a primary care practice with coordination/referral to community supports (personal support, meals, transportation). In Québec three Health and Social Service Centres were selected including 7) one network in a highly urban environment; 8) one network in a less densely populated urban environment; and 9) one rural network. These centres include one or more community health centres and long term care homes and either include or link to hospital care primary healthcare clinics, rehabilitation care, and private residential care. Each of the Québec cases share an organizational and governance structure, similar referral approaches, a common screening tool and intake process, and similar linkages to community services and other medical providers; the volume and types of care provided within the lead organization varies by geography. (see Breton et al., 2017) [34].

### Comparative Policy Analysis

Phase One also included an extensive comparative policy analysis designed to describe the institutional arrangements and policy environment for ICBPHC within Ontario, Québec, and New Zealand. From this we are able to better understand the policy arrangements that condition the development and implementation of ICBPHC as well as the historical trajectory of primary health care reform at a macro-system level. In New Zealand this includes reference to the Treaty of Waitangi. The analysis draws on literature, official documents, legislation, websites and publications of government agencies, professional organizations and other relevant institutions, and key informant interviews with system-level planners, policy-makers, health organisation managers and health professionals, and patients and family carers. Our analysis suggests that some jurisdictions have more favourable conditions for development of ICBPHC than others. Two key conditions are the organizational integration of relevant health and social sector organizations, and the range of policy levers available and used by governments. On both dimensions, the New Zealand environment appears to offer the largest scope, with Ontario’s environment significantly less conducive, and Québec situated in between. Nevertheless, in each jurisdiction there remain important institutional barriers to the implementation of policies that promote ICBPHC (see Tenbensel et al., 2017) [35].

### Empirical Comparison of Primary Care Performance

A third part of Phase One involved analysis of recent surveys of general practitioners and their patients that the research teams had undertaken in New Zealand and Canada, as part of a multi-country study that aimed to evaluated the quality, costs and equity of primary care in 34 countries [36]. The focus of these analyses is on how particular features of ICBPHC (for example, co-location of practitioners or comprehensiveness of a service) impact on self-reported outcomes of general practitioners and their patients. Using data from 330 primary care practices in New Zealand and Canada, we found that as the number of non-physicians increased, so did the availability of special sessions/clinics for patients with diabetes, hypertension, and older persons. Co-location was also associated with the provision of more disease management programs, more specialized equipment, and more nursing services [37].

## Phase Two: Develop a Conceptual Framework

Translating innovative models of ICBPHC requires understanding the attributes and workings of each model, the context in which it was developed, and the context into which a successful model might be spread. To achieve this, the second phase of the research involves multi-method case studies of each of the selected exemplar models encompassing macro (system), meso (organization and provider) and micro (patient and family carer) levels. Data collection is organised around four groups of expert informants: policy makers and system experts, organization managers, service providers, and patients and family carers. Data collection is now complete for this phase and analyses are under way.

### Policy level

At the policy level, we hypothesize that each of the case study models of ICBPHC care will be shaped by the organization or network of organizations in which the model of care is housed, a matrix of local or regional organizations that influence the operation of each model of care, the broader (current and historical) institutional arrangements and policy subsystems within which the model of care has developed, and the wider political, economic, and social or cultural context.

For each case study, we are collecting supporting documents relevant to the development and/or implementation of the model of care. We also undertake a series of 5–10 semi-structured, open-ended interviews with decision makers who have been directly engaged in the development and implementation of the model of care. In addition, we interview 10–15 people who are in leadership positions (current or past) within relevant policy subsystems at the regional or national level, or who for any other reason are considered to be interested and informed observers of the development of these innovative models of care. These include decision makers within government ministries, representatives of private provider associations, consumer advocates, leaders of professional groups and ethnic and indigenous minorities, and other key knowledge holders. Policy level interviews are semi-structured and included questions about how organizations became engaged in the development of ICBPHC, funding, approval and renewal processes, influences of the wider health system and policy initiatives including strategies, regulations/legislation, as well as recommended changes that would facilitate the implementation of ICBPHC.

### Organizational level

Similar to the policy level, we hypothesize that each of the case study models of ICBPHC would be shaped by the organization or network of organizations in which the model of care is situated. Prior research and evaluation of integrated care tends to focus on measuring the activities and outcomes of the intervention itself, and less emphasis has been placed on when and specifically how contextual factors matter. Therefore the focus of our organizational inquiry addresses “organizational context with an intention to describe the setting in which an integrated care intervention is implemented” [6] and to identify and measure organizational factors that are not a direct part of the intervention [7], such as governance structures, leadership approach, and organizational culture. To conduct the comparative case studies, we developed a case study guide and standardized tools for collecting comparable data on contextual factors across care providers, settings, and studies. (see Evans et al., 2017) [38].

Our organizational level data collection include interviews and surveys with approximately 10 organizational leaders at each of our case studies. The organizational interviews make use of our Context and Capabilities for Integrating Care (CCIC) framework that includes seven factors related to basic structures, seven related to people and values, and four related to processes [39]. From these 18 different factors related to context and capabilities, interviewees selected up to six factors and discussed how these factors affected the implementation of the integrated care model and commented on why other factors were not as important. Survey questionnaires were used to assess the degree to which organizations demonstrate performance on all factors in the CCIC framework.

### Provider level

Provider interviews are also used to uncover the extent to which, and the mechanisms by which, care is integrated for patients and family carers within each case. In particular our approach evaluates the extent to which the interventions leveraged the six elements of the Chronic Care Model. We hypothesize that effective models of care will have strong basis in all six elements. Provider questions were adapted from the Assessment of Chronic Illness Care [40,41]. Adaptation was necessary because the ACIC was developed for use in a team focus-group interview, whilst we used individual interviews in our case studies. Up to 15 direct care providers including front line health professionals and other health workers were selected from those delivering care as part of the ICBPHC model. Questions in the provider interviews ask about the extent to which and how the model achieved each of the six elements.

### Patient and Carer level

Patient engagement was given considerable importance in this research program. The key feature underlying all of our case studies was a focus on integrated and person-centred care for older persons with complex health needs. We explicitly engaged patients as part of the study team activities, including the development of terms of reference for undertaking selected case studies and as participants in the team itself. Health consumers and patients participated in questionnaire development and piloting advising on the types, language, structure and order of questions in the patient and family carer questionnaires. They also endorsed the inclusion of “Hua Oranga”, a research instrument that is underpinned by “Te Whare Tapu Wha” a well-established Māori health model (see Sheridan et al., 2017 in this collection) [42]. The New Zealand team included a researcher consumer (with an academic appointment) and senior consumer advocates who have senior health system governance and indigenous academic roles A patient and a carer also participated in the research team in Canada (see Hanson and Hanson 2017 in this collection) [43].

Our interviews with patients and family carers are conducted separately and selection of patients and carers was mostly not linked. (i.e., there were only a few patient-family carer dyads) Our target sample for patients and carers respectively was 15 per case. Patients and carers are identified and approached by the clinical or administrative teams at each site to ascertain whether they would be willing to be approached by a study team member. A study team member then contacts the patient or carer to explain the study and obtain consent in their preferred language (English, Māori, Cantonese, Mandarin). For consenting patients an in-person interview at a location of their convenience is arranged. For most, it is in their home. Interviews with patients include information on personal details, health conditions, and general well-being (physical, mental, spiritual and family), then explore engagement with primary health care providers including accessibility, the quality of the relationship and degree of shared decision-making using primarily structured survey questions [44], and then type and use of social agencies, participation in cultural and community activities and finally material standard of living. Family carer interviews include similarly structured survey questions including the Carer Reaction Assessment Scale [45], Hua Oranga [46], Cultural Justification for Caregiving Scale [47], as well as detailed questions about the type and extent of support provided to patients in addition to questions about the carer’s goals, general wellbeing, relationships with the person being cared for and their family, and interactions with members of the care team.

### Analysis

Our data collection results in an extensive set of up to 50 interviews per case plus policy-level interviews resulting in up to nearly 200 interviews in each jurisdiction. Interviews are first transcribed verbatim and entered into NVIV0 11 software [48], a data management software that supports qualitative data management, analysis and audit. Codes are then developed for each research perspective (policy, organizational, provider, patient, family carer) based on the contents of the transcripts themselves, the concepts from the frameworks from which the interviews were derived as well as emerging insights. The principles for coding across jurisdictions is to set common ‘parent’ codes for the entire project within each perspective and allow for local case and within jurisdiction variations in ‘child’ codes. Data definitions are common across jurisdictions.

Survey data are summarized with descriptive statistics and compared across case studies, and variability in measures across cases will also be explored where appropriate. Qualitative interview data and documents are analyzed using qualitative methods including the use of inductive thematic approaches [49,50]. Data will be integrated following a case-oriented approach, including legitimating the data and research findings and interpreting the mixed methods research findings [51,52]. These analyses will be used to address several questions that speak to the broader overarching research question guiding this study.

### Developing an integrated conceptual framework

The goal of this program is to develop a framework that incorporates the policy and community-context, organizational and provider configurations, interactions and adaptations that were required to enable innovative ICBPHC models to be implemented successfully in each jurisdiction.

Following the guidelines for integrating data in a mixed methods approach [51,52], the data will be combined with the use of a data integration table. In this process we will identify the attributes of the models and factors that affected the implementability and list these in rows of the table. For the columns of the table we will list target outcomes achieved by the models from a system perspective and from the patient and caregiver perspective using attributes of population health, patient experience, equity, and cost. The results of the analysis around specific issues and topics will be integrated to address the questions about what factors affected the degree of integration, achievement of elements of the Chronic Care Model, implementability and sustainability of the model and the outcomes achieved, and by what mechanisms.

A staged process will be used to populate a data integration table [51,52]:

1. *Data reduction* – The dimensionality of the data will be reduced to meet the needs of this synthesis through thematic analysis of the qualitative data collected, and by applying descriptive analysis to the quantitative data collected.
2. *Data display* – We will develop matrices, charts, graphs, lists and narratives to describe the qualitative data, and tables/graphs to display the quantitative data. These will be populated in the appropriate parts of the above table. This approach follows recommended approaches to integrate data for a mixed methods analysis [51,52] For example, charts and lists to describe qualitative data about the organizational factors that affect program uptake will be placed in the same cell as quantitative tables of data that relates to the number of patients engaged in the program over time, and the extent of cross organizational interaction over time.
3. *Data consolidation* involves combining and interpreting quantitative and qualitative data to create new or consolidated variables and themes. These are the variables/themes that will explain how the implementation factors affect the outcomes. This interpretative phase will be done in workshops with the researchers, including patients and consumer advocates, and key informants from the case-study models. The outcome of the process will be the identification and ranking of the factors that aided/inhibited implementation of the initiatives examined. This process will identify the factors that need to be considered when either scaling up the initiatives further locally, or transferring the initiatives to other contexts, or more generally for the scaling-up and spread of other promising approaches and models of integration (in phase 3).

From the data consolidation phase we will have identified key factors that affect the implementation pathway. This will then be used to populate the conceptual framework – an intermediate theory that explains how to implement successful ICBPHC models for older adults with complex care needs. We will workshop the integrated findings of Phase 2 with decision-makers to discuss the framework and constituent factors identified from our analysis of the 9 cases as well as the nature of the relationships between the factors. Given that this is a theory-building phase, this process will be interpretative and have a number of cycles of refinement. The outcome will be the framework itself, and a number of testable propositions.

## Phase Three: Apply the Framework in an Evaluation of Scale and Spread

In the third phase of the research program, the conceptual framework will be used to prospectively study and evaluate the scale and spread of a specific existing ICBPHC model in a real-world instance of change in at least one jurisdiction. We will work closely with individuals from policy, organization, provider and patient and carer levels in this collaboration. Implementation of the scale and spread will be informed by the findings of Phases 1 and 2 of our program, and propositions drawn from the conceptual framework will be evaluated in terms of the interaction between complex contexts and interventions in the scaling up and spread of innovation with a prospective implementation design. Case study methods will be used, enhanced by methods such as interrupted time series analysis of policy-relevant outcomes, including patient and family carer perspectives and experiences, and avoidable hospitalizations. In this phase, methods will be used as suggested by the United Kingdom’s Medical Research Council [53], and Pawson and Tilley’s approach to realist evaluation [54,55], explicitly acknowledging that interventions are likely to work better when adapted to local contexts. Administrative data will be observed over a period of at least 2 years prior to, and up to two years after the implementation of models in order to judge the effects of implementation.

## The Research Team

We approach this program of research with an experienced team of investigators with expertise required to study the multiple dimensions involved in implementing successful ICBPHC interventions. The study team members include experts representing disciplines of health policy, organizational behavior and change management, health economics, quality and performance measurement, epidemiology, ethics, and includes clinical expertise in primary health care, mental health, geriatrics, nursing, physiotherapy, home and social care, or are decision-makers with leadership roles in implementing changes in the health system. The team is enhanced by engagement with patient and family carers, academic consumer advocates and indigenous advisors.

## Discussion

Understanding the complex interaction between context and model implementation is at least as important as understanding the attributes and effectiveness of innovative models of ICBPHC for older adults with chronic conditions. The beneficial outcomes of this research program will be the development of a comprehensive conceptual framework to guide and evaluate the implementation and spread of innovative ICBPHC delivery models. New knowledge will be created about the transfer of innovation from one jurisdiction to another (inter and intra-nationally) including a measurement system and pathway of implementation of innovative ICBPHC models.

One challenge to our program is the study of implementation with a cross-sectional design. The process and journey of implementation of each program has taken place over years. Our approach is to explicitly ask questions in our interviews about the history and current progress toward implementing integrated care. We are also able to assess differences in the depth of integration and the strength of integrating mechanisms vis-a-vis the maturity and age of programs.

The approaches used to analyse the rich and extensive qualitative data will vary by specific research questions. The unique opportunity in this program is to allow not only for implementation of integrated care to be examined from multiple perspectives (from policy through to patient and carers) but to integrate these perspectives within and across cases and jurisdictions. Although initial coding will be developed within each perspective, themes that arise from one perspective can be examined within the data from other perspectives. By nature we expect the overlap of themes to be closest between ‘neighboring’ perspectives who interact with each other most (e.g. policy and organization, patient and provider), we will look explicitly for common themes for example regarding how information technology is used and implemented from the policy makers through to the carers, and how funding and costs related to the integrated care are perceived from each perspective. The six essential components of the CCM will also be explored across all cases and perspectives to evaluate the extent to which these are activated and enabling features for implementing integrated person-centered care for older adults with complex health needs.

Managing this international network of researchers who share common aspirations while varying in their priorities, brings its own challenges. For example, our approaches to equity and consumer involvement in research differed in emphasis between our researchers and jurisdictions despite a collective concern about enduring inequalities that affect peoples’ lives [42]. Sharing data could also come with considerable risks. Researchers’ proprietary views of collected data, familiarity with and preference for their disciplinary conceptualizations and approaches to analysis, and focused engagement in portions of a large project are all risks in achieving the full value of this collaboration. While creating opportunities for integration of contrasting world views, we are challenged to leverage the disciplinary strength of the team. We are taking a number of steps to mitigate these risks including the development and use of an explicit authorship policy and a common shared secure data repository in which all study data are stored. Research teams in all of the three jurisdictions meet four times each year by videoconference, and once every 18 months in person. In addition, individual team members are in regular contact with their counterparts in other jurisdictions by email and videoconference. Regular team meetings are viewed as essential for maintaining a common understanding of the aims of process of the research program, for aligning data collection and analysis processes, and for maintaining the general quality of the research programme. These are explicit strategies to enhance engagement and productivity across the team. Distributed leadership, initially with cross-jurisdictional representation within each perspective and then based on leadership for manuscripts with open invitations for participation across the entire team will enable local innovations and accelerate productivity.

## Conclusion

Our program of research is based on rich disciplinary strengths and knowledge of theory within each of the perspectives but the project outcomes will be enhanced by interdisciplinary participation and partnership with consumers in analysing and interpreting our results. Data collected in this study for each perspective draw on the latest theories and practices and have been used in various configurations in prior research. The depth within each perspective must be combined within a context that allows a broad view of the program of research. Uzzi and colleagues analysed over 17.9 million articles in the Web of Science and found that the propensity for highly cited papers (one measure of impact) was sharply elevated when combinations of prior work were anchored in disciplinary theory while mixing together multiple conventions in combinations rarely seen together [56]. We are aiming high with the potential implications of our research program in terms of our ability to change the way that integrated care is thought about and implemented internationally. We will engage directly with our partners and consumers in the dissemination of findings to local communities. The impact for our research will be measured in the improved care and experience of vulnerable individuals with complex health needs, of their carers and whānau (i.e., extended family), as well as improved provider and organizational outcomes.

## References

[1] Starfield, B. Point: The Changing Nature of Disease: Implications for Health Services. Medical Care. 2011; 49(11): 971–2. DOI: 10.1097/MLR.0b013e318233a0c1 22002639

[2] Bishop, B. The National Strategy for an Ageing Australia: Healthy Ageing: Discussion Paper Sydney, Australia: Commonwealth of Australia; 1999.

[3] Gething, L. We’re Growing Older Too: Quality of Life and Service Provision Issues for People with Long Standing Disabilities who are Ageing Sydney, Australia: University of Sydney; 1999.

[4] Ham, C. The ten characteristics of the high-performing chronic care system. Health Economics, Policy and Law. 2010; 5(1): 71–90. DOI: 10.1017/S1744133109990120 19732475

[5] Kodner, DL. Whole-system approaches to health and social care partnerships for the frail elderly: an exploration of North American models and lessons. Health & Social Care in the Community. 2006; 14(5): 384–90. DOI: 10.1111/j.1365-2524.2006.00655.x 16918830

[6] Wagner, EH, Austin, BT and Korff, MV. Organizing care for patients with chronic illness. The Milbank Quarterly. 1996; 74(4): 511–44. DOI: 10.2307/3350391 8941260

[7] de Bruin, SR, Versnel, N, Lemmens, L, Molema, C, Schellevis, F, Nijpels, G, et al. Comprehensive care programs for patients with multiple chronic conditions: a systematic literature review. Health Policy. 2012; 107(2–3): 108–45. DOI: 10.1016/j.healthpol.2012.06.006 22884086

[8] Coleman, K, Austin, BT, Brach, C and Wagner, EH. Evidence on the Chronic Care Model in the New Millennium. Health Affairs. 2009; 28: 75–85. DOI: 10.1377/hlthaff.28.1.75 19124857 PMC5091929

[9] Minkman, M, Ahaus, K and Huijsman, R. Performance improvement based on integrated quality management models: what evidence do we have? International Journal for Quality in Health Care. 2007; 19(2): 90–104. DOI: 10.1093/intqhc/mzl071 17277010

[10] Ouwens, M, Wollersheim, H, Hermens, R, Hulscher, M and Grol, R. Integrated care programmes for chronically ill patients: a review of systematic reviews. International Journal for Quality in Health Care. 2005; 17(2): 141–6. DOI: 10.1093/intqhc/mzi016 15665066

[11] Upshur, R and Tracy, C. Chronicity and complexity: facing the challenges of chronic disease in primary care. Canadian Family Physician. 2008; 54: 1655–8. 19074692 PMC2602629

[12] Hariharan, J, Tarima, S, Azam, L and Meurer, J. Chronic Care Model As a Framework to Improve Diabetes Care at an Academic Internal Medicine Faculty-Resident Practice. The Journal of Ambulatory Care Management. 2014; 37(1): 42–50. DOI: 10.1097/JAC.0000000000000007 24309394

[13] Fens, M, van Heugten, CM, Beusmans, G, Metsemakers, J, Kester, A and Limburg, M. Effect of a Stroke-Specific Follow-Up Care Model on the Quality of Life of Stroke Patients and Caregivers: A Controlled Trial. Journal of Rehabilitation Medicine. 2014; 46(1): 7–15. DOI: 10.2340/16501977-1239 24241508

[14] Lemmens, KMM, Lemmens, LC, Boom, JHC, Drewes, HW, Meeuwissen, JAC, Steuten, LMG, et al. Chronic care management for patients with COPD: a critical review of available evidence. Journal of Evaluation in Clinical Practice. 2013; 19(5): 734–52. 22133473 10.1111/j.1365-2753.2011.01805.x

[15] Parsons, M, Senior, H, Kerse, NM-hC, Jacobs, S and Vanderhoorn, S. Should Care Managers for Older Adults Be Located in Primary Care? A Randomized Controlled Trial. Journal of the American Geriatrics Society. 2012; 60(1): 86–92. DOI: 10.1111/j.1532-5415.2011.03763.x 22239292

[16] Hébert, R, Raîche, M, Dubois, M-F, Gueye, NR, Dubuc, N, Tousignant, M, et al. Impact of PRISMA, a coordination-type integrated service delivery system for frail older people in Quebec (Canada): a quasi-experimental study. Journal of Gerontology:Social Sciences. 2009; 65B(1): 107–18. DOI: 10.1093/geronb/gbp027 19414866

[17] Beland, F, Bergman, H, Lebel, P and Dallaire, L. Integrated Services for Frail Elders (SIPA): A Trial of a Model for Canada. Canadian Journal on Aging/La Revue Canadienne du Vieillissement. 2006; 25(1): 25–42. DOI: 10.1353/cja.2006.0019 16770746

[18] Beland, F, Bergman, H, Lebel, P, Clarfield, AM, Tousignant, P, Contandriopoulos, AP, et al. A system of integrated care for older persons with disabilities in Canada: Results from a randomized controlled trial. Journals of Gerontology Series a-Biological Sciences and Medical Sciences. 2006; 61(4): 367–73. DOI: 10.1093/gerona/61.4.367 16611703

[19] Wodchis, W, Dixon, A, Anderson, G and Goodwin, N. Integrating care for older people with complex needs: key insights and lessons from a seven-country cross-case analysis. International Journal of Integrated Care. 2015; 15(6). DOI: 10.5334/ijic.2249 PMC4628509 26528096

[20] Peek, CJ, Cohen, DJ and deGruy, FV. Research and Evaluation in the Transformation of Primary Care. American Psychologist. 2014; 69(4): 430–42. DOI: 10.1037/a0036223 24820691

[21] Aarons, GA, Hurlburt, M and Horwitz, SM. Advancing a Conceptual Model of Evidence-Based Practice Implementation in Public Service Sectors. Adm Policy Ment Health. 2011; 38: 4–23. DOI: 10.1007/s10488-010-0327-7 21197565 PMC3025110

[22] Damschroder, LJ, Aron, DC, Keith, RE, Kirsh, SR, Alexander, JA and Lowery, JC. Fostering implementation of health services research findings into practice: a consolidated framework for advancing implementation science. Implementation Science. 2009; 4(50). DOI: 10.1186/1748-5908-4-50 PMC2736161 19664226

[23] Berwick, DM. The science of improvement. JAMA. 2008; 299(10): 1182–4. DOI: 10.1001/jama.299.10.1182 18334694

[24] Lanham, HJ, Leykum, LK, Taylor, BS, McCannon, CJ, Lindberg, C and Lester, RT. How complexity science can inform scale-up and spread in health care: Understanding the role of self-organization in variation across local contexts. Social Science & Medicine. 2013; 93(0): 194–202. DOI: 10.1016/j.socscimed.2012.05.040 22819737

[25] WHO. Innovative Care for Chronic Conditions: Building Blocks for Action World Health Organization; 2002.

[26] Rafferty, AE, Jimmieson, NL and Armenakis, AA. Change readiness: A multilevel review. Journal of Management. 2013; 39(110). DOI: 10.1177/0149206312457417

[27] Tabak, RG, Khoong, EC, Chambers, DA and Brownson, RC. Bridging Research and Practice Models for Dissemination and Implementation Research. Am J Prev Med. 2012; 43(3): 337–50. DOI: 10.1016/j.amepre.2012.05.024 22898128 PMC3592983

[28] Meyers, DC, Durlak, JA and Wandersman, A. The Quality Implementation Framework: A Synthesis of Critical Steps in the Implementation Process. Am J Community Psychol. 2012; 50: 462–80. DOI: 10.1007/s10464-012-9522-x 22644083

[29] Bertram, RM, Blasé, KA and Fixsen, DL. Improving Programs and Outcomes: Implementation Frameworks and Organization Change. Research on Social Work Practice; 2014.

[30] Kaplan, HC, Brady, PW, Dritz, MC, Hooper, DK, Linam, WM, Froehle, CM, et al. The influence of context on quality improvement success in health care: A systematic review of the literature. Milbank Quarterly. 2010; 88(4): 500–59. DOI: 10.1111/j.1468-0009.2010.00611.x 21166868 PMC3037175

[31] Rycroft-Malone, J, Seers, K, Chandler, J, Hawkes, CA, Crichton, N, Allen, C, et al. The role of evidence, context, and facilitation in an implementation trial: implications for the development of the PARIHS framework. Implement Sci. 2013; 8(28). DOI: 10.1186/1748-5908-8-28 PMC3636004 23497438

[32] Ullrich, PM, Sahay, A and Stetler, CB. Use of implementation theory: A focus on PARIHS. Worldviews on Evidence-Based Nursing. 2013; 1–9. 24103045 10.1111/wvn.12016

[33] Kuluski, K, Sheridan, N, Kenealy, T, Breton, M, McKillop, A, Shaw, J, et al. “On the Margins and Not the Mainstream:” Case Selection for the Implementation of Community based Primary Health Care in Canada and New Zealand. International Journal of Integrated Care. 2017; 17(2): 15 DOI: 10.5334/ijic.2501 28970756 PMC5624111

[34] Breton, M, Steele Grey, C, Sheridan, N, Shaw, J, Parsons, J, Wankah, P, et al. Implementing Community Based Primary Healthcare for Older Adults with Complex Needs in Quebec, Ontario and New-Zealand: Describing Nine Cases. International Journal of Integrated Care. 2017; 17(2): 12 DOI: 10.5334/ijic.2506 28970753 PMC5624082

[35] Tenbensel, T, Miller, F, Breton, M, Couturier, Y, Morton-Chang, F, Ashton, T, et al. How do Policy and Institutional Settings Shape Opportunities for Community-Based Primary Health Care? A Comparison of Ontario, Québec and New Zealand. International Journal of Integrated Care. 2017; 17(2): 13 DOI: 10.5334/ijic.2514 28970754 PMC5624106

[36] Schafer, WLA, Boerma, WGW, Kringos, DS, De Maeseneer, J, Gress, S, Heinemann, S, et al. QUALICOPC, a multi-country study evaluating quality, costs and equity in primary care. BMC Family Practice. 2011; 12: 115 DOI: 10.1186/1471-2296-12-115 22014310 PMC3206822

[37] Rumball-Smith, J, Wodchis, WP, Koné, A, Kenealy, TW, Barnsley, J and Ashton, T. Under the same roof: co-location of practitioners within primary care is associated with specialized chronic care management. BMC Family Practice. 2014; 2(15): 149 DOI: 10.1186/1471-2296-15-149 PMC4171578 25183554

[38] Evans, JM, Grudniewicz, A, Steele Gray, C, Wodchis, WP, Carswell, P and Baker, GR. Organizational Context Matters: A Research Toolkit for Conducting Standardized Case Studies of Integrated Care Initiatives. International Journal of Integrated Care. 2017; 17(2): 9 DOI: 10.5334/ijic.2502 PMC5624120 28970750

[39] Evans, JM, Grudniewicz, A, Baker, GR and Wodchis, WP. Organizational Capabilities for Integrating Care: A Framework for Improvement. International Journal of Integrated Care. 2016; 16(3): 15 DOI: 10.5334/ijic.2416 PMC5388061 28413366

[40] Wagner, EH, Glasgow, RE, Davis, C, Bonomi, AE, Provost, L, McCulloch, D, et al. Quality improvement in chronic illness care: a collaborative approach. Jt Comm J Qual Improv. 2001; 27(2): 63–80. DOI: 10.1016/S1070-3241(01)27007-2 11221012

[41] Bonomi, AE, Wagner, EH, Glasgow, RE and VonKorff, M. Assessment of Chronic Illness Care (ACIC): A Practical Tool to Measure Quality Improvement. Health Services Research. 2002; 37: 791–820. DOI: 10.1111/1475-6773.00049 12132606 PMC1434662

[42] Sheridan, N, Kenealy, T, Stewart, L, Lampshire, D, Robust, TT, Parsons, J, et al. When Equity is Central to Research: Implications for Researchers and Consumers in the Research Team. International Journal of Integrated Care. 2017; 17(2): 14 DOI: 10.5334/ijic.2512 PMC5624117 28970755

[43] Hanson, F and Hanson, R. Reflections from a Patient and Carer on Involvement in Research and Integrating Care in the Health System. International Journal of Integrated Care. 2017; 17(2): 16 DOI: 10.5334/ijic.3088 28970757 PMC5624061

[44] Glasgow, RE, Wagner, EH, Schaefer, J, Mahoney, LD, Reid, RJ and Greene, SM. Development and validation of the Patient Assessment of Chronic Illness Care (PACIC). Med Care. 2005; 43(5): 436–44. DOI: 10.1097/01.mlr.0000160375.47920.8c 15838407

[45] Given, C, Given, B, Stommel, M, Collins, C, King, S and Franklin, S. The Caregiver Reaction Assessment (CRA) for caregivers to persons with chronic physical and mental impairments. Research in Nursing & Health. 1992; 15: 271–83. DOI: 10.1002/nur.4770150406 1386680

[46] Durie, MH and Kingi, TKR. A Framework for Measuring Maori Mental Health Outcomes A report prepared for the Ministry of Health. 1997; Palmerston North, New Zealand: Massey University.

[47] Dilworth-Anderson, P, Goodwin, PY and Williams, SW. Can Culture Help Explain the Physical Health Effects of Caregiving Over Time Among African American Caregivers? The Journal of Gerontology: Social Sciences. 2004; 59B(3): S138–S45. DOI: 10.1093/geronb/59.3.S138 15118019

[48] QSR International. NVivo qualitative data analysis software [cited 2016 July 26]. Available from: http://www.qsrinternational.com/.

[49] Braun, V and Clarke, C. Using thematic analysis in psychology. Qualitative Research in Psychology. 2006; 3(2): 77–101. DOI: 10.1191/1478088706qp063oa

[50] Richie, J and Spencer, L. Qualitative Data Analysis for Applied Policy Research In: Bryman A and Burgess RG (ed.), Analyzing Qualitative Data. 1994; 173–94. London: Routledge. Taylor and Fracis Books Ltd DOI: 10.4324/9780203413081_chapter_9

[51] Onwuegbuzie, A and Leech, N. Linking research questions to mixed methods data analysis procedures. The Qualitative Report. 2006; 11: 474–98.

[52] Onwuegbuzie, A, Slate, J, Leech, N and Collins, K. Mixed data analysis: advanced integration techniques. International Journal of Multiple Research Approaches. 2009; 3: 13–33. DOI: 10.5172/mra.455.3.1.13

[53] Medical Research Council. A Framework for Development and Evaluation of RCTs for Complex Interventions to Improve Health, United Kingdom: Medical Research Council; 2000.

[54] Greenhalgh, T, Humphrey, C, Hughes, J, Macfarlane, F, Butler, C and Pawson, R. How Do You Modernize a Health Service? A Realist Evaluation of Whole-Scale Transformation in London. Milbank Quarterly. 2009; 87(3): 391–416. DOI: 10.1111/j.1468-0009.2009.00562.x 19523123 PMC2881448

[55] Pawson, R and Tilley, N. Realistic Evaluation London: Sage; 1997.

[56] Uzzi, B, Mukherjee, S, Stringer, M and Jones, B. Atypical combinations and scientific impact. Science. 2013; 342(6157): 468–72. DOI: 10.1126/science.1240474 24159044

